# A Novel -72 (T→A) *β*-Promoter Mutation Causing Slightly Elevated HbA_2_ in a Vietnamese Heterozygote

**DOI:** 10.1155/2017/4537409

**Published:** 2017-04-19

**Authors:** Monica Pirastru, Paolo Mereu, Chau Quynh Nguyen, Nhan Viet Nguyen, Thang Duy Nguyen, Laura Manca

**Affiliations:** ^1^Dipartimento di Scienze Biomediche, Università di Sassari, Sassari, Italy; ^2^Hematology Department, Hue University, Hue, Vietnam; ^3^Genetic Department, Hue University, Hue, Vietnam

## Abstract

We report a novel *β*^+^-thalassemia mutation found in a Vietnamese family. The molecular defect T→A lies at -72 of the *β*-globin gene promoter, within the conserved CCAAT box. The index case was a 5-year-old child having red blood cells indices close to normal and slightly increased level of HbA_2_ (3.96%). The expression of the mutated *β* allele was inferred by luciferase reporter assay in K562 cells. The *β* -72 determinant is the eighth *β*-thalassemic mutation identified in Vietnam and it was not previously reported in any population. The absence of homozygous or compound heterozygous states did not allow us to precisely predict either its clinical impact or its relevance in management programs. Our results further underline the importance of identifying and characterizing new or rare *β*^+^-thalassemic alleles in carrier screening and prenatal diagnosis.

## 1. Introduction

*β*-Thalassemia is a heterogeneous genetic disease associated with defective expression of the *β*-chain of human hemoglobin (Hb). So far, more than 300 mutations [1] which affect almost every known stage of *β*-globin gene expression resulting in a reduction (*β*^+^) or complete absence (*β*^0^) of *β*-chain synthesis from the affected allele have been described (HbVar database: http://globin.cse.psu.edu). Several *β*-thalassemia defects come from point mutations that involve three highly conserved promoter sequence motifs. They are located between positions -26 and -105 with respect to the Cap site and promote the *β*-globin gene expression by means of the interaction with the *β* Locus Control Region (LCR) and several* trans*-acting factors [2].

In Vietnam, the carrier rate for *β*-thalassemia ranges from 1.5% to 25% depending on the ethnic population groups. The first study for *β*-thalassemia was performed in the North of Vietnam [3]. Later, the spectrum of *β*-globin mutations has been investigated in Ho Chi Minh City, South Vietnam [4, 5]. Preliminary data concerning the central area of the country were recently published [6, 7]. To date, seven *β*-thalassemia mutations have been identified in the Vietnamese population. All of them were already described in other countries of the Southeast Asia with different incidence. Interaction between these mutations and the rather common Hb E leads to a variety of thalassemia syndromes, in particular to the severe forms of homozygous *β*-thal and Hb E-*β*-thal diseases. Nonetheless, epidemiological data is still insufficient and fragmented.

Here we describe a novel *β*^+^ promoter mutation -72 (T→A) identified during a screening program for hemoglobinopathies (Hbpathies) carried out as part of the ongoing cooperation between the Universities of Hue, Vietnam, and Sassari, Italy. The mutation was found in a 5-year-old Vietnamese child and in two of his relatives. The functional effect of this mutation was evaluated by luciferase reporter assays.

## 2. Materials and Methods

The study was conducted on a Vietnamese child, suspected to be a carrier of *β*-thalassemia, and his relatives.

### 2.1. Hematology and Hemoglobin Analysis

Hematological parameters were measured by the Blood Analyzer SYSMEX KX-21 and SYSMEX 800i (Japan Care, Co., Ltd.).

Hb tetramers separation was performed by cation-exchange high-performance liquid-chromatography (CE-HPLC), with the Chromsystems Instruments & Chemicals (GmbH, Germany), and by isoelectric focusing [8].

### 2.2. Molecular Analysis

Genomic DNA was isolated from peripheral blood leukocytes using the Invisorb® Spin Blood Midi Kit, according to the manufacturer's instructions (STRATEC Biomedical AG, Birkenfeld, Germany).

### 2.3. Multiplex Ligation-Dependent Probe Amplification (MLPA) Analysis

MLPA analysis using SALSA MLPA probemix 140 HBA (MRC-Holland, Amsterdam, Netherlands) was carried out to exclude the coinheritance of *α*-thalassemia. Ligation and amplification reactions were performed on a GeneAmp® PCR System 2700 thermal cycler (Applied Biosystems, Foster City, CA, USA). MLPA products were separated by ABI PRISM 3130 Genetic Analyzer (Applied Biosystems, Foster City, CA, USA) and quantified as already described [9].

### 2.4. PCR and Sequencing Analysis

In order to clone the fragment containing the *β*-globin gene promoter in a pBluescript II SK (pSK) vector, *β*_KpnI and *β*_XhoI primers, engineered to contain both* Kpn*I and* Xho*I restriction site, respectively, were used.

The entire proband's *β*-globin gene was amplified and sequenced. Polymerase Chain Reactions (PCR) were performed as described in Table 1 by using the primers shown in Table 2. All amplified products were electrophoresed through a 1–1.2% agarose, 1x TAE, and ethidium bromide stained gel at 7.5 volts/cm for 45′ in the presence of a molecular weight marker. DNA was recovered from agarose by means of the Montage Gel Extraction Kit (Merck Millipore, Darmstadt, Germany). The purified fragments were sequenced by terminator chemistry (BigDye Terminator v3.1 Cycle Sequencing Kit, Applied Biosystems, Foster City, CA, USA). Reaction mix was purified through the Sigma Spin Postreaction Clean-Up Columns (Sigma-Aldrich, Saint Louis, MO, USA) and subjected to capillary electrophoresis on an ABI PRISM 3130 Genetic Analyzer (Applied Biosystems, Foster City, CA, USA). Another Plasmid Editor APE (http://biologylabs.utah.edu/jorgensen/wayned/ape/) was used to align the obtained sequences with the reference (AC #: U01317).

### 2.5. Plasmid Constructions and Mutagenesis

The PCR fragment containing the *β*-globin gene promoter was digested and inserted into the* Kpn*I and* Xho*I restriction sites of pSK vector. By cloning, two different constructs were obtained: the pSK_*β*WT containing the wild type promoter and the pSK_*β*-72 containing the -72 (T→A) mutated one.

Two mutated constructs were also generated by site-directed mutagenesis starting from the pSK_*β*WT: the pSK_*β*-87 and the pSK_*β*-71, containing the -87 C→G and -71 C→T mutation, respectively. The mutagenesis reaction was performed in a volume of 50 *μ*l and consisted in 1x Pfu Buffer, 20 ng of pSK_*β*WT, 200 *μ*M of dNTPs, 125 ng of each primer (Table 2), and 3 U of Pfu. Thermocycle parameters were 30′′ at 95°C; 18 cycles of 30′′ at 95°C, 1′ at 55°C, and 90′′/kb at 72°C; additional final extension was added (10′ at 72°C). PCR products were subsequently incubated 1 h at 37°C with 3 U of* Dpn*I to digest the methylated parental plasmid.

To perform the luciferase assay, wild type and mutated promoters were transferred into the pGL2-Basic Luciferase Reporter Vector (Promega, Madison, WI, USA) containing the HS2 region. We generated the recombinant plasmid pGL2-HS2 inserting the HS2-locus control region into the pGL2-Basic. The HS2 fragment was amplified (Table 1) using the primers HS2s and HS2as (Table 2) and inserted into the* BamH*I and* Sal*I restriction sites of pGL2-Basic.

All the plasmid constructions were verified by automated sequencing after transformation of CaCl_2_ competent DH5*α* bacteria and purification of a 50 ml culture by PureLink® HiPure Plasmid Midiprep Kit (Life Technologies, Thermo Fisher Scientific, Waltham, MA, USA).

### 2.6. Cell Culture and Luciferase Assays

K562 cells were grown in RPMI 1640 GlutaMAX™ medium (Gibco, Thermo Fisher Scientific, Waltham, MA, USA), containing 10% fetal bovine serum, 100 mg of streptomycin, and 100 U/ml of penicilin at 5% CO_2_ and 37°C.

Dual Luciferase Assay (Promega, Madison, WI, USA) was performed: the* Renilla *luciferase expression vector pRL-TK (Promega, Madison, WI, USA) was cotransfected with the pGL2 recombinant plasmids (1 : 20 pRL : pGL2) as an internal control.

Transfection was performed with the Lipofectamine LTX reagent (Life Technologies, Thermo Fisher Scientific, Waltham, MA, USA) according to the manufacturer's protocol; briefly 500 ng of total plasmid DNA was added to 0.5 *μ*l of Plus in 100 *μ*l of Opti-MEM serum and antibiotic-free medium (Gibco, Thermo Fisher Scientific, Waltham, MA, USA) and incubated for 5′ at room temperature; 1.25 *μ*l of LTX was then added. After a 30′ incubation, the mixture was added to 1 · 10^5^ cells/500 *μ*l in a 24-well plate and incubated for 24 h at 37°C. The luciferase activity was measured through a luminometer (Victor X5, PerkinElmer, Waltham, MA, USA). The experiment was performed independently in quadruplicate and the data were expressed with a mean ± standard deviation (SD). Differences between WT and mutant groups were analyzed using Student's *t*-test; *P* values less than 0.05 were considered statistically significant.

## 3. Results

The propositus showed a mild anemia with an increased HbA_2_ level of 3.96%. His relatives have been also analyzed: the father and grandfather showed similar HbA_2_ levels (4.01 and 3.82%, resp.), whereas the other family members displayed normal hematological parameters and Hb components. The hematology data of the proband and his relatives are presented in Table 3.

**Figure 1:**
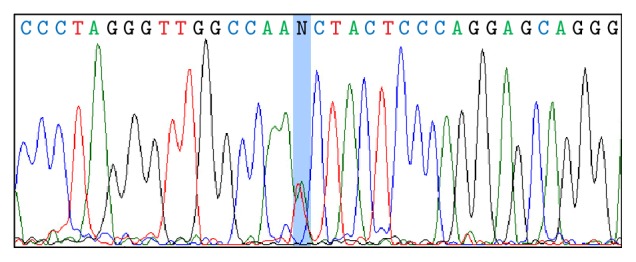
Nucleotide sequencing of the *β*-globin gene promoter showing the T→A heterozygosity at position -72 from the Cap site, in the CCAAT box.

The index case was found to have a novel to literature T→A mutation at position -72 from the Cap site, located in the CCAAT box of the *β*-promoter region (Figure 1). Sequencing revealed that his father and grandfather shared the same *β*^−72^*β* genotype.

Four common polymorphic sites [codon 2 (CAC→CAT), IVS-II-16 (C*→*G), IVS-II-74 (G*→*T), IVS-II-666 (T*→*C)] were also detected and the following configuration CAT, G, T, C, was found* in cis* to the -72 mutation. The coupling of specific *β*-globin gene mutations with neutral changes has been widely described [10, 11]; it allowed the detailed characterization of chromosome regions in which mutant *β*-globin genes reside.

**Figure 2:**
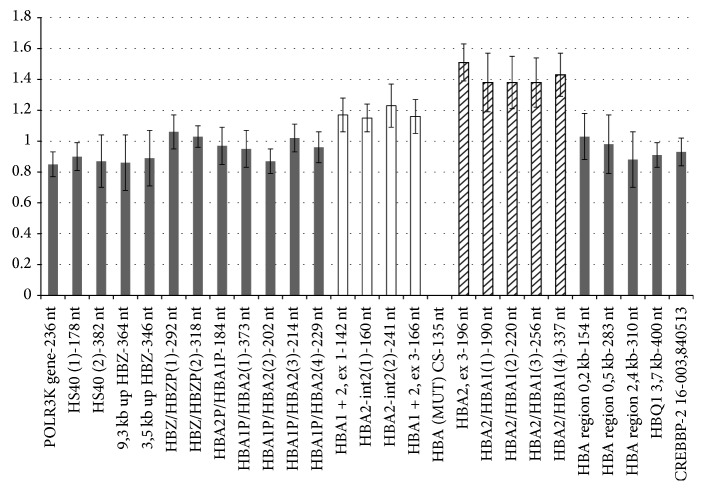
Multiplex ligation-dependent probe amplification performed on DNA from proband's father using SALSA MLPA kit P140-B4 HBA. The horizontal axis shows the MLPA probes arranged according to chromosomal location. The vertical axis shows the normalized probe ratio. White and hatched columns show increased height ratios (~1.2 versus 1 and 1.4 versus 1, resp.). These probe ratios are expected for a heterozygous triplication (product description probemix P140 HBA, MRC-Holland). The HBA(MUT)CS-135 nt probe is specific for the presence of the Constant Spring mutation and does not generate a signal in negative samples.

No alteration was observed in the HBA cluster of the proband and his grandfather, whereas a triplicated HBA cluster, that seems to have no effect on the phenotype, was pointed out in the proband's father. The resulting bar chart has been shown in Figure 2. The presence of Hb Constant Spring was excluded in all carriers.

Luciferase assay was performed using the pGL2-HS2-*β*-87 and pGL2-HS2-*β*-71 constructs as positive control. Both -87 and -71 have been already described as *β*-thalassemia mutations. The *β* -87 C→G allele is a mild transcriptional mutant described in Mediterranean countries [12]. It alters the proximal CACCC box, a crucial element for the expression of the *β*-globin gene. Homozygotes [13] or compound heterozygotes for *β* -87 and for severe *β*-thalassemia mutations [14] are affected with thalassemia intermedia (TI). The -71 C→T mutation occurs one nucleotide immediately downstream of the core CCAAT sequence. Based on hematological phenotypes in simple heterozygotes, as well as in compound heterozygotes with HbS [*β*6(A3)Glu>Val], the mutation was assigned as a mild *β*^+^-thalassemic allele [15].

**Figure 3:**
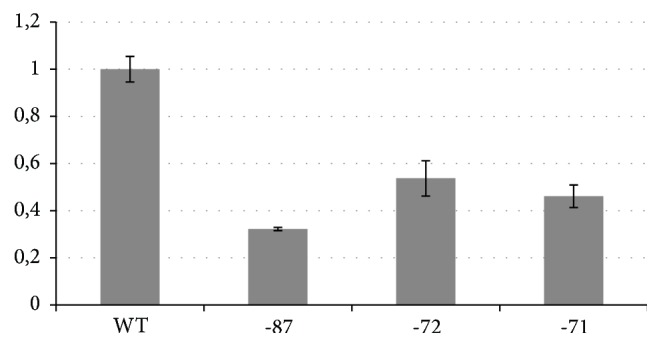
Relative luciferase activity of mutants *β*-globin promoters. WT: wild type promoter, -87: promoter containing the -87 C→G mutation, -72: promoter containing the -72 T→A mutation, -71: promoter containing the -71 C→T mutation.

In our experiment the expression of the three mutant vectors was compared with the pGL2-HS2-*β*-WT, which was considered to have 100% activity. The results of the transfection studies are summarized in Figure 3. Luciferase activities of the mutant controls (-87 and -71) were significantly decreased, demonstrating that the system is able to reproduce a downregulation of the *β*-globin gene promoter in vitro. Relative luciferase activities of -87, -72, and -71 mutated promoters were 32.3 ± 0.7%, 53.7 ± 7.5%, and 46.1 ± 4.8%, respectively. These results clarify that -72 mutation, as well as that described for -87 and -71, is a mild *β*-thalassemic allele.

## 4. Discussion 

The thalassemias, together with sickle cell disease, are the world's most common form of inherited anemia. After 60 years of significant progress, the management of these conditions still depends on supportive care, regular lifelong blood transfusions, and iron chelation. Cure is possible only in the limited case of a patient having an HLA-identical donor. Approximately 80% of the annual births of babies with severe conditions occur in developing and low-income countries, many of which have extremely limited facilities for their control and management. It is therefore important to accurately identify carriers of these disorders and offer the option of preventive measures by prenatal diagnosis to couples at risk of having a child with severe disease. Routine diagnosis of *β*-thalassemia trait is based on microcytic parameters and/or elevated levels of HbA_2_ (≥3.5). However, a reliable diagnosis can only be achieved by DNA analysis.

The Vietnamese population is ethnically highly heterogeneous and the spectrum of *β*-thalassemia alleles is slowly defining [3–7, 16]. On the whole, six mutations of the *β*^0^ and a mutation of the *β*^+^ type have been identified and observed with distinct incidence in the different areas. The most commons are the frameshift mutations at codons 41/42 (-TCTT) and the nonsense mutation at codon 17 (A→T). Other mutations, although less frequent, are the -28 (A→G), the frameshift mutations at codons 71/72 (+A) and at codon 95 (+A); the IVS-I-1 (G→T); and the IVS-II-654 (C→T). Among these *β*-thalassemic alleles, the -28 A→G is the only mutant detected at *β*-promoter [4, 5]. The *β* -28 determinant was also reported in the two countries neighboring Vietnam, China, and Thailand [17, 18]. This variant occurs in the conserved sequence of the TATA box, located at position -30 to -26 from the Cap site and has been introduced into the HbVar database (http://globin.cse.psu.edu/) as *β*^+^-thalassemic allele. Indeed, the mild thalassemic phenotype observed in compound heterozygotes *β*^E^/*β*^−28^ from Thai population has indirectly indicated that the -28 mutation is a mild thalassemic allele [18].

In this study, a novel promoter mutation of -72 (T→A) within the conserved CCAAT box of *β*-globin gene has been identified in heterozygous state. The proband and his two relatives carrying the same mutation showed almost normal mean corpuscular volume (MCV) and mean corpuscular hemoglobin (MCH) level and slightly elevated HbA_2_. Similar hematological parameters and HbA_2_ levels were previously described in heterozygous state for the mild *β*^+^-thalassemic allele -73 (A→T) [19]. This mutation, immediately upstream of the -72, also occurs in the CCAAT box and leads to a slightly reduced *β*-globin mRNA level of 19.35% compared with normal individuals.

Our in vitro experiments in K562 cells show that the transcriptional activity of the mutated promoter is roughly half that of the wild type promoter. This finding suggests that the -72 mutation can be classified as a *β*^+^-thalassemic allele. Association of *β*^+^-thalassemia with *β*^0^ or *β*^E^ mutations results in a markedly heterogeneous hematological picture, ranging in severity from that of the *β*-thalassemia carrier state to that of thalassemia major (TM) [20]. Furthermore, it has been noted that, even in the case of mild disease, *β*-TI patients may still suffer from many complications including a hypercoagulable state and subsequent thrombotic events [21].

## 5. Conclusions 

The here described -72 determinant is the second *β*^+^-thalassemic mutation identified at the promoter level in the Vietnamese population. This mutation, observed in the heterozygous state, is only associated with slightly elevated HbA_2_. Nevertheless a mild phenotype of a resulting association between the -72 mutation and another severe form of *β*-thalassemia cannot be taken for granted.

The ability to predict phenotype from genotype has important implications for the screening of *β*-thalassemia carriers, for genetic counseling and prenatal diagnosis, and for planning the appropriate treatment regimen.

Our results further underline the importance of identifying and characterizing new or rare *β*^+^-thalassemic alleles in carrier screening and prenatal diagnosis in order to reduce the burden of thalassemias, avoid unnecessary transfusions in TI, and start early transfusions in TM.

## Figures and Tables

**Table 1:** Tabulated details of PCR conditions.

Primer pair	Target region	MgCl_2_ (mM)	dNTPs (*μ*M)	Thermal conditions^*∗*^
*β*1-*β*2	HBB (5′ UTR to exon 2)	3.5	250	(94°C^1 min^-65°C^45 sec^-72°C^1 min^) × 35 cycles
*β*7-*β*8	HBB (exon 2 to IVS 2)	3.0	250	(94°C^1 min^-60°C^1 min^-72°C^1 min^) × 35 cycles
*β*9-*β*10	HBB (IVS 2 to 3′ UTR)	1.5	250	(94°C^1 min^-55°C^1 min^-72°C^1 min^) × 35 cycles
*β*_KpnI-*β* _XhoI	HBB (promoter)	4.0	300	(94°C^1 min^-66°C^1 min^-72°C^4 min^) × 35 cycles
HS2_BamHI-HS2_SalI	*β*-LCR (HS2)	4.0	200	(94°C^1 min^-64°C^1 min^-72°C^2 min^) × 35 cycles

^*∗*^Each thermal profile was preceded by a denaturation of 94°C for 3 min and followed by an additional extension of 74°C for 4 min.

**(a):** 

Primer pairs used for standard PCR and sequencing			
Primer code	Sequence (5′ to 3′)	Nucleotide position (#U01317)	Product size
*β*1(F)	GCCAAGGACAGGTACGGCTGTCATC	61997–62021	706 bp
*β*2(R)	CCCTTCCTATGACATGAACTTAACCAT	62676–62702	
*β*7(F)	TCCTGATGCTGTTATGGGCAA	62469–62489	923 bp
*β*8(R)	AAAAGCAGAATGGTAGCTGGA	63371–63391	
*β*9(F)	AAAAACTTTACACAGTCTGCC	62935–62955	966 bp
*β*10(R)	ATTAGCTGTTTGCAGCCTCA	63881–63900	

**(b):** 

Primer pairs used for cloning and sequencing			
Primer code	^*∗*^Sequence (5′ to 3′)	Nucleotide position (#U01317)	Product size
*β*_KpnI(F)	ggtaccATCCAGTTTCTTTTGGTTAACCT	60678–60700	1505 bp
*β*_XhoI(R)	ctcgagTCTGTTTGAGGTTGCTAGTGAACAC	62158–62182	
HS2_BamHI(F)	ggatccTAAGCTTCAGTTTTTCCTTAGT	8485–8506	740 bp
HS2_SalI(R)	gtcgacTAGATCTGACCCCGTATGTGAGCAT	9200–9224	

**(c):** 

Primer pairs used for site-direct mutagenesis	
Primer code	°Sequence (5′ to 3′)
-87G(F)	CTCACCCTGTGGAGCCACAC***G***CTAGGGTTGGCCAATCTAC
-87G(R)	GTAGATTGGCCAACCCTAG***C***GTGTGGCTCCACAGGGTGAG
-71T(F)	CTAGGGTTGGCCAAT***T***TACTCCCAGGAGCAGG
-71T(R)	CCTGCTCCTGGGAGTA***A***ATTGGCCAACCCTAG

# = Genbank accession number.

^*∗*^Restriction sites are in lowercase.

°Mutated nucleotides are in boldface and italic. The F and R primer sequences are complementary to each other.

**Table 3:** Hematology, *β*-genotype, and globin clusters arrangement for proband and his family members.

Samples	Hb (g/dl) ^*∗*^12–15	HbA2 (%) ^*∗*^≤3.5	MCV (fl) ^*∗*^80–100	MCH (pg) ^*∗*^28–32	*β*-Genotype	HBB cluster	HBA cluster
Proband	10.8	3.96	83	26.3	*ββ* ^−72^	Normal	Normal
Father	14.1	4.01	96.5	30.5	*ββ* ^−72^	Normal	Triplicated
Mother	12.0	2.94	96.8	29.7	Normal	ND	ND
Sister	12.1	2.84	85.1	26.9	Normal	ND	ND
Grandfather	13.6	3.82	95.4	29.5	*ββ* ^−72^	Normal	ND
Grandmother	11.9	2.69	97.5	30	Normal	ND	ND

^*∗*^Reference values.
